# A case of pediatric serum sickness like reaction (SSLR) after a 2-month re-exposure to amoxicillin

**DOI:** 10.1186/s13223-024-00887-7

**Published:** 2024-04-01

**Authors:** Devyani Bakshi, Xinxin Tang, Susan Waserman

**Affiliations:** Division of Clinical Immunology and Allergy, Department of Medicine, McMaster University, Hamilton, Ontario Canada

**Keywords:** Serum sickness, Amoxicillin, Serum sickness like reaction, Pediatric

## Abstract

**Background:**

Serum-sickness like reactions (SSLRs) to amoxicillin have been documented in the medical literature. Beta-lactams are important and commonly used medications especially in the pediatric population. Often, SSLRs present within days of and during first exposure/ingestion to the offending agent. We described a unique case of a 4-year-old boy who presented with symptoms of amoxicillin SSLR following his second course of amoxicillin with only 2 months and 10 days between his second and first course.

**Case presentation:**

A 4-year-old boy presented to hospital with a pruritic rash on day 7 of a 10-day course of amoxicillin for otitis media accompanied by fever (38.7 degrees Celsius). On day 7 of his second course of amoxicillin, which was separated from his first course by only 2 months and 10 days, his mother noticed erythematous, raised, pruritic lesions with central clearing on his sternum. He presented to the ED with emesis, progression of the rash to his torso, back, legs, and face, hypotension, angioedema, and joint pain. His bloodwork demonstrated a leukocytosis of 18.6 × 10^9^ g/L with neutrophilic predominance and thrombocytosis with a platelet count of 653 × 10^9^ g/L. He was treated with 5 mg oral cetirizine daily and 1 mg/kg oral prednisone which improved his rash and angioedema. He was managed with up to 4 times the usual dose of cetirizine. He was assessed in our outpatient clinic as an outpatient and penicillin skin testing was unremarkable. A diagnosis of a probable SSLR to amoxicillin was made.

**Conclusion:**

We report an unusual presentation of SSLR following re-exposure to amoxicillin. Our case highlights that patients with previous asymptomatic exposure to amoxicillin can develop SSLR with repeat exposure. Although it is not uncommon for children to develop amoxicillin SSLRs after previous exposure to the drug, this case is unique because of its short time course of 2 months and 10 days months between drug courses. Penicillins are commonly used in the pediatric population. Therefore, it is important to correctly characterize adverse drug reactions to broaden our understanding of SSLRs, prevent unnecessary avoidance of the triggering agent, and improve patient management.

## Background

Serum sickness like reactions (SSLRs) are non-immune complex mediated reactions which are characterized by rash, fever, and polyarthralgias [1]. SSLRs are triggered by viral infections, vaccines, and drugs, with many reported cases of SSLRs to amoxicillin [2, 3]. Some case reports and literature reviews have suggested that amoxicillin SSLRs make up approximately 4–87% of all adverse drug reactions associated with amoxicillin [2, 4]. The prevalence of amoxicillin induced SSLRs is variable given the differential diagnosis is broad and often misdiagnosed as other pathologies such as viral exanthem, erythema multiforme, urticarial vasculitis, etc. SSLRs are more common in the pediatric population and often occur within 5–10 days of exposure when caused by beta-lactams [1]. There have been multiple cases of pediatric SSLRs following re-exposure to amoxicillin, however, to our knowledge, the time between re-exposure has not been well studied and 2 months and 10 days between two courses of amoxicillin is the shortest reported interval between development of an SSLR [2, 5].

SSLRs, unlike serum sickness reactions, do not involve the formation of immune complexes and are not IgG mediated [1]. Despite the prevalence of pediatric amoxicillin SSLRs, there are no validated diagnostic criteria and due to heterogeneity of presentation, diagnosis is not always straightforward.

## Case presentation

We report a case of an otherwise healthy 4-year-old boy who presented to hospital with a pruritic rash on day 7 of a 10-day course of amoxicillin for otitis media accompanied by fever (highest 38.7 degrees) and chills. Prior to his hospitalization, the patient had received a 7-day course of amoxicillin for a dental infection without any issues. His first course of amoxicillin was separated from his second by 2 months and 10 days.

**Fig. 1:**
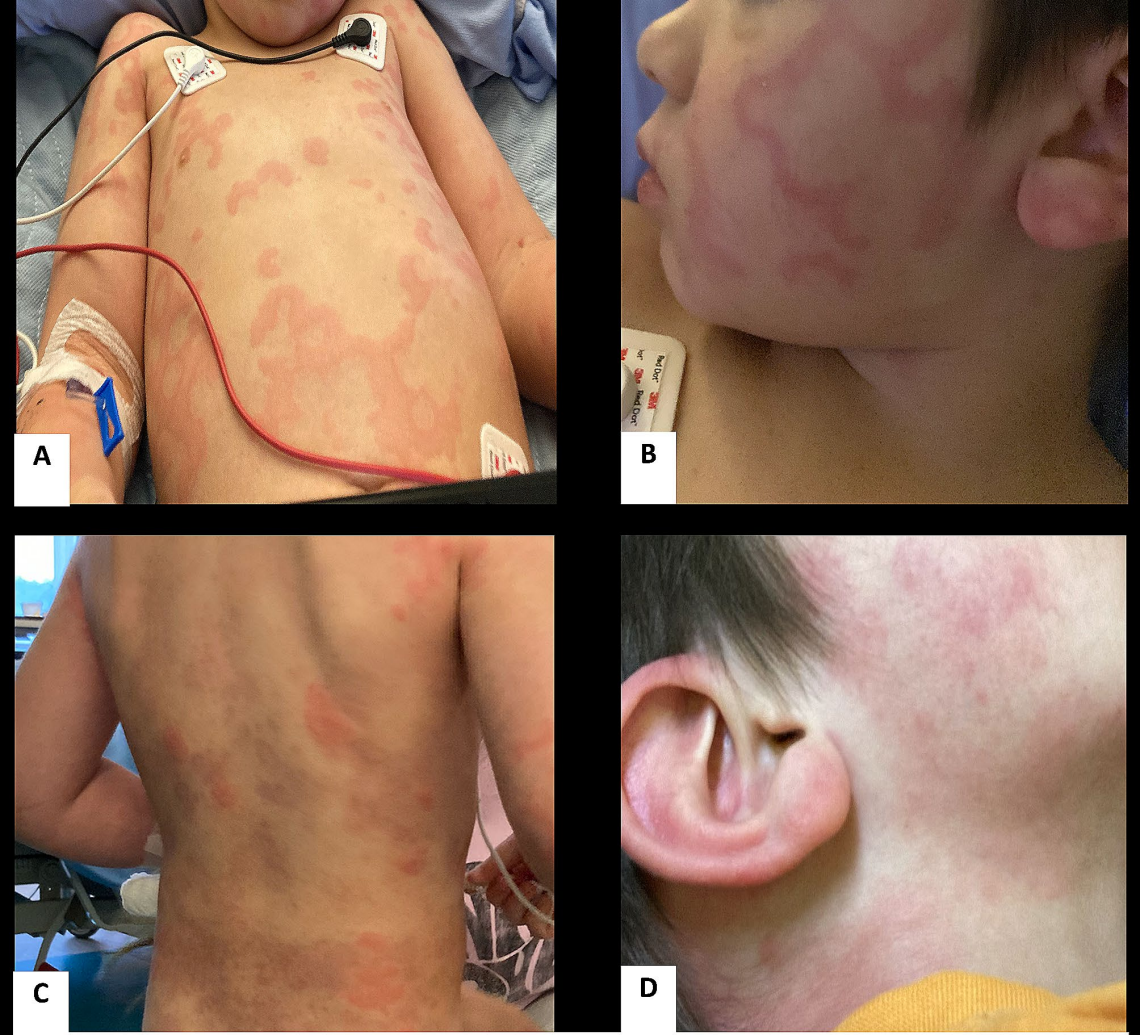
Papular, erythematous pruritic patches across the patient’s torso, chest, arms (**A**), cheeks, lateral neck (**B** and **D**), and across the back.

On day 7 of his second course of amoxicillin for otitis media, his mother noticed erythematous, raised, pruritic lesions with central clearing on his sternum (Fig. 1). They called his family doctor who prescribed 10 mg of oral Rupatadine and instructed them to monitor for signs of improvement. He had taken 20 mg of Rupatadine in total; however, despite treatment, his rash spread to his legs and became more pruritic that same evening, prompting his visit to our children’s hospital.

While in the emergency room, he had 3 episodes of non-bilious, non-bloody emesis and spreading of the rash to his torso, back, bilateral legs, and face (Fig. 1). He also became hypotensive (systolic blood pressure of 43 mmHg at its lowest) and developed left hand and elbow angioedema which prevented him from opening his hand. In addition, he reported difficulty walking with pain in his right knee, likely due to joint pain.

He was given one round of 0.15 mg intramuscular epinephrine and intravenous fluids (500 mL of Ringer’s Lactate (RL) followed by 70 mL/hr of maintenance RL for 16 h). His blood pressure (BP) increased to 102/50 after his IV fluid bolus and epinephrine, however his rash remained unchanged. On physical examination, he had palpable, erythematous, blanchable, urticarial-like lesions with central clearing across his forehead, cheeks, upper arms, and thighs. He also had perioral and periorbital angioedema. No synovitis or lymphadenopathy was appreciated on examination. His abdomen was soft, non-tender to palpation, and his chest was clear. He was admitted to hospital with a presumptive diagnosis of anaphylaxis.

His complete blood count demonstrated an elevated leukocytosis of 18.6 × 10^9^ g/L (5-14.5 × 10^9^ g/L) with neutrophil predominance, normal hemoglobin at 132 g/L (115–135 g/L), and thrombocytosis with a platelet count of 653 × 10^9^ g/L (150–400 × 10^9^ g/L). Other than an alanine aminotransferase (ALT) level of 18 U/L (24–45 U/L), liver enzymes were normal. Electrolytes were normal, as was serum tryptase. His creatinine and eGFR were normal at 33 µmol/L (16–35 µmol/L) and 86 mL/min/1.73m^2^. His C-reactive protein was not elevated (< 5.0 mg/L) on admission, but 2 days after his initial presentation, was elevated at 37.9 mg/L. Other initial investigations revealed a normal chest x-ray, and abdominal ultrasound. His urinalysis showed trace ketones but was otherwise normal. Viral infection workup including Epstein-Barr virus (EBV) PCR, cytomegalovirus IgM serology, Herpes Simplex 1 and 2, and Varicella Zoster was negative. Nasopharyngeal viral swab was negative on two occasions but positive at the time of discharge for norovirus, which family was informed about post-discharge. Blood cultures were negative as was throat culture for Group A Streptococcus. His C3 and C4 complement levels were normal at 1.15 (0.81–1.57 g/L) and 0.16 g/L (0.13–0.39 g/L), respectively. An ultrasound of his left arm revealed an anechoic lesion measuring 2.6 × 2.3 × 0.6 cm, limited to soft tissue and consistent with lymphatic malformation.

While hospitalized, he was treated with 5 mg oral cetirizine daily and 1 mg/kg oral prednisone which helped relieve his rash and elbow/hand angioedema, but he developed angioedema of his eyelids and lips a day after his initial presentation whilst in hospital. His rash had progressed to involve his arms and legs, as well as abdomen, back, and buttocks (genitals were spared) the day of admission but began to clear after day 2 of admission while he was on the antihistamines and steroids. He was assessed by the allergy and immunology consult service who recommended up to 4 times the licenced dose of second-generation antihistamines PRN for symptom relief, and that he be further assessed in our outpatient clinic, a few weeks after discharge. Intradermal penicillin skin testing was performed using histamine 1.0 mg/mL as a positive control 0.1 cc, saline buffer as a negative control 0.2 cc, penicillin G (10,000 units/mL) 0.2 cc, and benzylpenicilloyl polylysine 0.2 cc. His penicillin test was negative, defined as a bleb less than 3 mm and no reaction greater than the negative control (0 mm).

The differential diagnosis at the time included drug related IgE-mediated anaphylaxis versus viral infection -induced urticaria and infection related symptoms versus SSLR to amoxicillin. Anaphylaxis was less likely given he had the amoxicillin for 7 days prior to any symptoms and eventual negative penicillin skin tests. He was discharged home from hospital 6 days after his initial presentation.

A diagnosis of SSLR to amoxicillin was made. His parents elected to forgo a graded oral challenge at the time given his degree of illness and prolonged admission. At this time though not definitively proven, SSLR was felt to be the most likely diagnosis.

## Discussion and conclusions

Beta-lactams, especially, amoxicillin, are commonly used drugs in the pediatric population due to their efficacy, safety, and low cost [6]. Therefore, it is important to correctly characterize adverse drug reactions because of their implications for future use. SSLRs are commonly mistaken for viral exanthem, urticaria, erythema multiforme or other adverse drug reactions [4]. Since SSLRs are a clinical diagnosis without validated diagnostic criteria, blood work and laboratory assessments are limited, making diagnosis more challenging [6]. Children under 4 years of age are mostly affected [2, 6–8].

In contrast to serum sickness reactions, the pathophysiology of SSLRs is poorly understood, and different causative agents may cause disease via different cellular mechanisms. For example, an in vitro study by *Kearns et al.* demonstrated direct toxicity of cefaclor on lymphocytes [9]. Alternatively, Penicillin G or its metabolites may act as a haptens, binding to endogenous serum proteins and increasing their immunogenicity, which may have been the case for our patient [7]. Nevertheless, the presentation of SSLR can be like classic SS and presents with the same triad of fever, arthralgia, and rash [8]. Symptoms tend to be less severe than classic serum sickness, and patients often develop low-grade fevers [7, 9]. Symptoms develop within 5–10 days although the timeline is variable [7]. Common triggers of SSLR include cefaclor, penicillins and other β-lactam antibiotics, and viral infections [8].

Currently, there is no standard management, treatment, and prognosis for amoxicillin SSLRs [2]. SSLRs are frequently conditions which resolve on their own with supportive care [2]. The use of antihistamines, non-steroids anti-inflammatory (NSAID) medications, and oral corticosteroids remains controversial without strong quality of evidence [2, 8]. Oral steroids have been shown to decrease time to symptom resolution but often duration of treatment is uncertain [8]. In addition to the diagnostic uncertainty in the management of patients with amoxicillin SSLRs, there is also no standardization and heterogeneity with regards to future repeat use of penicillins in these patients. Factors that are associated with a subsequent reaction to the offending agent in patients include male sex, reaction within 7 days of first dose of antibiotic, amoxicillin as the offending drug, and parental history of drug allergy [8]. This subsequent reaction often presents as macular/popular or urticarial-like rash in patients [8]. Previously, common practice involved avoidance of the offending agents, but labelled penicillin allergies are often associated with increased morbidity, longer hospitalizations, and the use of less targeted antibiotics [8]. In a cohort study by *Colli et al.* who used graded oral challenges (GOCs) in children presenting with SSLRs, the GOCs were safe and helped differentiate between penicillin induced SSLRs as opposed to viral exanthems [8]. Repeat challenges in the form of GOCs may help provide more diagnostic clarity and help de-label penicillin allergies.

This case report describes an example of SSLR. However, unlike typical SSLRs or serum sickness reactions, in our case, the patient also experienced significant hypotension. This could be explained by concurrent gastrointestinal viral illness, poor hydration, and subsequent systemic vasodilation in repones to his illness. Viral exanthem can be difficult to delineate from SSLR symptoms given the similarities in presentation [8].

We report an unusual presentation of SSLR after re-exposure to amoxicillin. We present a boy who developed clinical symptoms 7 days into his second course of amoxicillin. His first and second course of amoxicillin were only separated by 2 months and 10 days. Clinicians commonly overlook and underdiagnose SSLRs to amoxicillin in the pediatric population due to lack of standardized diagnostic criteria [6]. Although there have been cases of pediatric amoxicillin SSLRs following re-exposure to the drug, to our knowledge, 2 months and 10 days is an unusually short duration between drug courses to develop such a reaction and has not yet been reported in the literature [2, 5]. Moreover, our case helps contribute to our understanding of SSLRs due to the variability in its presentation. Amoxicillin SSLRs are not always associated with joint pain, hypotension, and good response to antihistamines as seen in our patient [2]. More research into amoxicillin SSLRs in the pediatric population is needed to better understand these conditions and improve our ability to manage them. This will help reduce burden of illness, prevent unnecessary avoidance of the triggering agent, and improve patient management.

## Data Availability

Data sharing is not applicable to this article as no datasets were generated or analysed during the current study.

## Abbreviations

SSLRs: Serum sickness-like reactions
SS: Serum sickness
BP: blood pressure
RL: Ringer’s lactate
ALT: Alanine aminotransferase
EBV: Epstein-Barr virus
eGFR: Estimated glomerular filtration rate
GOC: Graded oral challenge
